# Down syndrome-associated haematopoiesis abnormalities created by chromosome transfer and genome editing technologies

**DOI:** 10.1038/srep06136

**Published:** 2014-08-27

**Authors:** Yasuhiro Kazuki, Yuwna Yakura, Satoshi Abe, Mitsuhiko Osaki, Naoyo Kajitani, Kanako Kazuki, Shoko Takehara, Kazuhisa Honma, Hirofumi Suemori, Satoshi Yamazaki, Tetsushi Sakuma, Tsutomu Toki, Ritsuko Shimizu, Hiromitsu Nakauchi, Takashi Yamamoto, Mitsuo Oshimura

**Affiliations:** 1 Department of Biomedical Science, Institute of Regenerative Medicine and Biofunction, Graduate School of Medical Science, Tottori University, 86 Nishi-cho, Yonago, Tottori 683-8503, Japan; Tel: +81-859-38-6219; 2 Chromosome Engineering Research Center (CERC), Tottori University, 86 Nishi-cho, Yonago, Tottori 683-8503, Japan; 3 Division of Pathological Biochemistry, Department of Biomedical Sciences, Faculty of Medicine, Tottori University, 86 Nishi-cho, Yonago, Tottori 683-8503, Japan; 4 Department of Embryonic Stem Cell Research, Institute for Frontier Medical Sciences, Kyoto University, 53 Kawahara-cho, Shogoin, Sakyo-ku, Kyoto 606-8507, Japan; 5 Division of Stem Cell Therapy, Center for Stem Cell Biology and Medicine, Institute of Medical Science, University of Tokyo, Tokyo 108-8639, Japan; 6 Department of Mathematical and Life Sciences, Graduate School of Science, Hiroshima University, Higashi-Hiroshima 739-8526, Japan; 7 Department of Pediatrics, Hirosaki University Graduate School of Medicine, 5 Zaifu-cho, Hirosaki 036-8562, Japan; 8 Department of Molecular Hematology, Tohoku University Graduate School of Medicine, Sendai 980-8575, Japan

## Abstract

Infants with Down syndrome (DS) are at a high risk of developing transient abnormal myelopoiesis (TAM). A *GATA1* mutation leading to the production of N-terminally truncated GATA1 (GATA1s) in early megakaryocyte/erythroid progenitors is linked to the onset of TAM and cooperated with the effect of trisomy 21 (Ts21). To gain insights into the underlying mechanisms of the progression to TAM in DS patients, we generated human pluripotent stem cells harbouring Ts21 and/or GATA1s by combining microcell-mediated chromosome transfer and genome editing technologies. In vitro haematopoietic differentiation assays showed that the GATA1s mutation blocked erythropoiesis irrespective of an extra chromosome 21, while Ts21 and the GATA1s mutation independently perturbed megakaryopoiesis and the combination of Ts21 and the GATA1s mutation synergistically contributed to an aberrant accumulation of skewed megakaryocytes. Thus, the DS model cells generated by these two technologies are useful in assessing how GATA1s mutation is involved in the onset of TAM in patients with DS.

Down syndrome (DS), or trisomy 21 (Ts21), is the most frequent live-born aneuploidy syndrome in humans^1^, and new-born infants with DS are at a high risk of developing transient abnormal myelopoiesis (TAM)^2^. In most cases, TAM resolves spontaneously within 3 months. However, DS-related acute megakaryoblastic leukaemia (DS-AMKL) subsequently develops within 4 years in approximately 20–30% of cases with a history of TAM^3^,^4^,^5^. Therefore, TAM has been considered as a preleukaemic stage. Acquired mutations in the N-terminal activation domain of the megakaryocyte transcription factor GATA1, leading to the expression of a GATA1 isoform (GATA1s), have been reported in DS-TAM and DS-AMKL^6^,^7^,^8^. Furthermore, it has been reported that DS-TAM is most likely caused by a combination of the single GATA1 mutation and constitutive Ts21, and DS-AMKL evolved from a TAM clone that acquired additional mutation(s)^9^. However, the precise mechanisms in the progression process have not been clarified yet.

Patient-derived pluripotent stem cells, including embryonic stem (ES) and induced pluripotent stem (iPS) cells, are important tools to model pathology^10^,^11^,^12^,^13^,^14^. Although in vitro studies using DS-ES and DS-iPS cells reproduced the haematopoietic abnormalities in DS^15^,^16^,^17^, DS-derived pluripotent stem cells with an acquired *GATA1* mutation have not been generated. In this study, we generated novel Ts21, GATA1s, and GATA1s/Ts21 human ES cells by combining chromosome transfer and genome editing technologies.

## Results and Discussion

**Figure 1:**
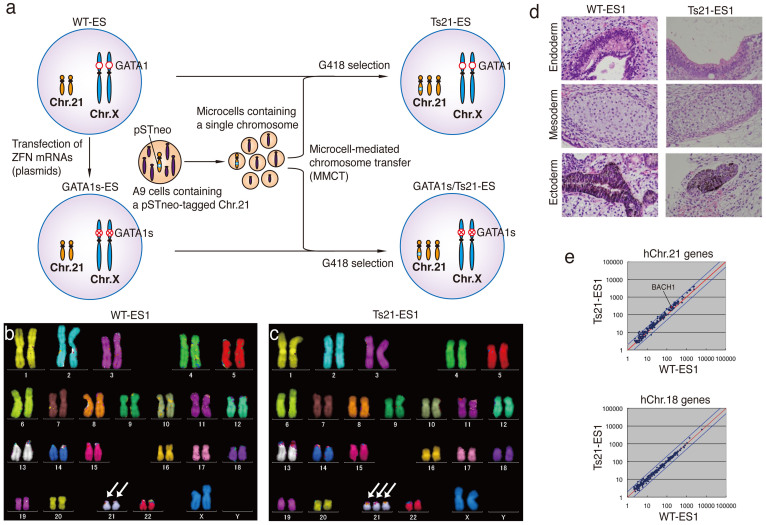
MMCT of hChr.21 into human ES cells. (a) Diagram of the generation of Ts21, GATA1s, and GATA1s/Ts21 in human ES cells. (b, c) mFISH analyses in WT-ES and Ts21-ES cells. Arrows indicate hChr.21. (d) Teratomas derived from WT-ES and Ts21-ES cells. Eight weeks after cell transplantation, structures originating from all 3 germ layers were found in the teratoma. (e) Microarray analyses of WT-ES and Ts21-ES cells. Representative comparison data of genes on hChr.21 (upper panel) and hChr.18 (lower panel) between WT-ES and Ts21-ES cells are shown. The dots between blue and red lines, and the dots on red line show genes with 2-fold differences in expression and equal expression, respectively. The red dot indicates *BACH1*, a representative gene from hChr.21 (upper panel).

A human chromosome 21 (hChr.21) was transferred to human ES cells via microcell-mediated chromosome transfer (MMCT)^18^. We previously generated a monochromosomal hybrid library in mouse A9 cells, which contained a single human chromosome^19^. DS model mice were generated by transferring an extra hChr.21 into mouse ES cells using the A9 library via MMCT^20^,^21^. Similarly, we generated human ES cells containing an extra hChr.21, creating Ts21. A pSTneo-tagged hChr.21 was transferred to human ES (KhES-1)-derived subclones (designated as WT-ES) via MMCT (Fig. 1a). Twelve G418-resistant clones from 3 independent experiments were obtained. Six clones contained an additional hChr.21 (Ts21), and 6 clones contained 2 additional copies of hChr.21 (tetrasomy 21) (Supplementary Fig. 1). Multicolour fluorescence in situ hybridisation (mFISH) analysis indicated that the hChr.21 was successfully transferred into wild-type (WT)-ES cells and that the karyotype was 47,XX,+21 (Fig. 1b, c). FISH analysis of the exogenous hChr.21 showed that the pSTneo-derived signal was in a single hChr.21 (Supplementary Fig. 2). To determine whether Ts21-ES cells could differentiate into all 3 embryonic germ layers, Ts21-ES lines were injected into testes of severe combined immunodeficiency (SCID) mice. Histological analyses revealed all 3 embryonic germ layers in all teratomas (Fig. 1d). Microarray analyses revealed that genes on hChr.21 in Ts21-ES cells were globally overexpressed, but gene expression from hChr.18 was comparable with that in WT-ES cells (Fig. 1e). These data suggest that the exogenous hChr.21 was successfully transferred to WT-ES cells and that the Ts21-ES cells have differentiation potential.

**Figure 2:**
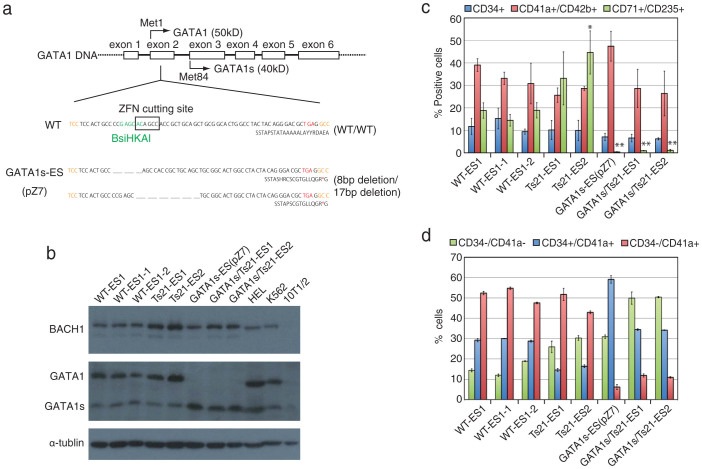
Characterisation of GATA1s-ES and GATA1s/Ts21-ES cells. (a) Sequence analyses of GATA1s-ES cells. One allele had an 8-bp deletion and the other had a 17-bp deletion; both resulted in a TGA stop codon in exon 2 of *GATA1*. The *GATA1* nucleotide (upper line) and amino acid (lower line) sequences are shown for WT-ES and GATA1s-ES cells. An asterisk shows the stop codon. (b) Western blot analyses of erythroid lineage-differentiated cells derived from WT-ES, WT-ES sublines, Ts21-ES lines, GATA1s-ES, and GATA1s/Ts21-ES lines. Anti-BACH1 was used to detect the gene-dosage effect on hChr.21. Anti-GATA1 recognised the C-terminus of both GATA1 and GATA1s protein. Anti-α-tubulin was used as an internal control. HEL cell lysate and K562 nuclear extract were used as positive controls. 10T1/2 whole cell lysate was used as a negative control. Cropped blots were used in this figure. Original full-length blots are shown in supplementary figure 12. (c, d) Haematopoietic differentiation analyses using WT-ES, WT-ES sublines, Ts21-ES lines, GATA1s-ES, and GATA1s/Ts21-ES lines. Data are the means of 3 independent experiments (±S.D.). The percentage of CD34+, CD41a+/CD42b+, and CD71+/CD235+ cells are shown in each differentiation stage (ES-sac (day 14), megakaryocyte (day 20), and erythroid (day 20)) (c). Statistical analyses were performed by comparison with WT-ES cells (WT-ES1, WT-ES1-1 and WT-ES1-2). *p < 0.05, **p < 0.01 by two-tailed Student's t test. The percentage of CD34-/CD41a-, CD34+/CD41a+ and CD34-/CD41a+ cells are shown in the megakaryocyte stage (d).

The *GATA1* mutation was generated via one of the genome editing technologies, zinc-finger nucleases (ZFNs), which were used previously to modify the endogenous genome of several species^22^. mRNAs or plasmids encoding a ZFN targeting exon 2 of *GATA1* DNA were transfected into WT-ES cells. A mutation detection assay (Cel1 assay) showed that 5 of 384 clones and 2 of 96 clones using the mRNAs and plasmids, respectively, were positive for the mutation (Supplementary Fig. 3). The mutation-positive mRNA-transfected clones were subcloned to reduce the possibility of heterogeneous populations. A restriction fragment length polymorphism (RFLP) assay using BsiHKAI enzyme showed that 1 (pZ7) of 19 clones (17 mRNA-transfected subclones and 2 plasmid-transfected clones) contained the different deletions in both alleles of exon 2 of *GATA1* (Supplementary Fig. 4); sequence analyses revealed that 2 clones (pZ19-2 and pZ28-5) contained heterozygous insertion/deletion (or deletion) in the *GATA1* gene and 1 clone (pZ7) contained different deletions (8 bp and 17bp) in both alleles, resulting in a premature TGA stop codon in exon 2 (Supplementary Figs. 5 and 6 and Fig. 2a). The clones with the premature stop codon in exon 2 had normal karyotypes (46,XX) and differentiation potential to 3 embryonic germ layers (Supplementary Figs. 7 and 8). The pZ7 clone (designated GATA1s-ES) with the deletions in both alleles of exon 2 of *GATA1* was used for further analyses.

An additional hChr.21 was transferred to GATA1s-ES cells via MMCT. Cytogenetic and histological analyses showed that the clones in the GATA1s genetic background contained Ts21, and 2 clones with Ts21 and GATA1s with differentiation potential to 3 embryonic germ layers in the teratoma were randomly selected for further analyses (designated GATA1s/Ts21-ES) (Supplementary Fig. 9). Microarray analyses revealed that genes on hChr.21 in GATA1s/Ts21-ES cells were globally overexpressed, but gene expression from hChr.18 was comparable with that in GATA1s-ES cells (Supplementary Fig. 10).

ES-sac–mediated in vitro haematopoietic differentiation analyses were performed (Supplementary Fig. 11). Western blot analyses of the erythroid lineage showed that a representative protein from hChr.21, BACH1^23^, was overexpressed in the Ts21-ES and GATA1s/Ts21-ES lines compared with the WT-ES and GATA1s-ES cells (Supplementary Fig. 12 and Fig. 2b). Western blot analyses using a GATA1 antibody to recognise the C-terminus of GATA1/GATA1s protein showed that full-length GATA1 protein was expressed in WT-ES and Ts21-ES lines, but not in GATA1s-ES and GATA1s/Ts21-ES lines (Supplementary Fig. 12 and Fig. 2b). Additionally, GATA1s protein was remarkably increased in the GATA1s-ES and GATA1s/Ts21-ES lines. These results were also confirmed in the megakaryocytic differentiation from the ES cell lines (data not shown). These data suggest that the protein expression pattern in the genetically engineered ES-derived haematopoietic cells was comparable to that of DS and DS-TAM patients^6^.

Flow cytometry analyses showed that the frequency of ES-sac–mediated erythroid (CD71+/CD235+) cells was higher in Ts21-ES cells than WT-ES cells. This is comparable to the results of a previous report by Chou et al., which demonstrated the enhanced erythropoiesis in iPS cells derived from Ts21 subjects^16^. In contrast, the frequency of erythroid cells in GATA1s-ES and GATA1s/Ts21-ES cells was significantly lower (Supplementary Fig. 13 and Fig. 2c). This is comparable to the results of previous reports by Hallanda et al. and Sankaran et al., which demonstrated the impaired erythropoiesis in the subjects with inherited *GATA1* mutations in the absence of Ts21^24^,^25^. Thus, the eyrhroid differentiation seems to be reciprocally affected by GATA1s or Ts21, and the effect of the GATA1s mutation overcomes the enhancing activity of erythropoiesis in Ts21-ES cells.

Curiously, we found that the population of ES-sac–mediated megakaryocytic (CD41a+/CD42b+) cells from GATA1s-ES cells was higher than that from WT-ES cells, although the further introduction of an additional hChr.21 into each cell line (WT-ES and GATA1s-ES cells) resulted in a slightly reducted frequency. (Supplementary Fig. 14 and Fig. 2c). Further analysis of the megakaryocytic differentiation showed that the ratio of immature (CD34+/CD41a+) to mature (CD34-/CD41a+) megakaryocytic cells derived from GATA1s-ES and GATA1s/Ts21-ES cells was significantly higher than that from WT-ES and Ts21-ES cells, respectively, suggesting that GATA1s disturbs the maturation of megakaryocytes and/or enhances the proliferation of immature megakaryocytes (Supplementary Fig. 15 and Fig. 2d). Furthermore, CD34-/CD41a− cells accumulated in Ts21-ES cell cultures with a reduced frequency of CD34+/CD41a+ cells, and the additional GATA1s mutation worsened the phenotype of Ts21 (Supplementary Fig. 15 and Fig. 2d). Consequently, the efficiency of CD41a+/CD42b+ cells in GATA1s/Ts21-ES cells was seemingly comparable to that in WT-ES cells. We concluded that Ts21 and GATA1s mutation differentially affect the megakaryocyte differentiation, and the combination of Ts21 and GATA1s mutations synergistically influences the process of megakaryocyte differentiation. Our in vitro differentiation system revealed for the first time that Ts21 disturbs the differentiation of megakaryocytes and further GATA1s mutation intricately perturbs the process of megakaryopoiesis in combination with increasing the dosage of genes located on hChr.21.

Taken together, our novel system combined MMCT and ZFN technologies to generate DS model cells. The combination of chromosome transfer and genome editing technologies could therefore enable the generation of in vitro chromosome abnormality syndrome models with multiple genetic alterations. Progression from TAM to DS-AMKL requires additional mutations in genes including cohesin/*CTCF*, *EZH2*, other epigenetic regulators, and *RAS*/signal transducing molecules^9^. However, the function or mechanism of each class of mutation on the leukaemogenesis remains uncertain. Our methods can aid in resolving these questions, because the desired mutations in addition to the GATA1s can be inserted to the GATA1s/Ts21-ES cells using genome editing technologies. Importantly, all of the developed ES cell lines were isogenic and genetically defined. MMCT using other chromosome donor A9 cells will enable the generation of pluripotent stem cell-derived models for different trisomy syndromes including Ts18 and Ts13 in the same genetic background. Human chromosomes can be efficiently modified in the homologous recombination-proficient chicken DT40 cells, which can be used as a shuttle system to transfer the modified chromosome to other cells^26^. Defined genomic regions can be also cloned into human artificial chromosomes^27^,^28^. Thus, the genes responsible for phenotypes may be identified by transferring the modified chromosome via MMCT.

## Methods

### Cell culture

Mouse A9 cells containing hChr.21 (A9(21-16)) that were used as fusion donors for chromosome transfer were established as described previously^19^. The A9(21-16) cells were maintained in Dulbecco's modified Eagle's medium plus 10% foetal bovine serum (FBS) with 800 μg/mL G418 (Promega, Tokyo, Japan). A human ES line, a KhES-1-derived subline^29^, was used following the human ES cell research guidelines of the Japanese government. Because the subline contained chromosomal abnormalities in chromosome 1q, the subline was further subcloned (designated as WT-ES1). WT-ES1 was used for MMCT and ZFN transfection experiments. WT-ES1 was further subcloned for the control cell lines (designated as WT-ES1-1 and WT-ES1-2). The parental human ES cell line and microcell hybrid clones were maintained on mitomycin C (Sigma-Aldrich, Tokyo, Japan)-treated Jcl:ICR (CLEA Japan, Tokyo, Japan) mouse embryonic fibroblasts as feeder layers in primate ES medium (ReproCell, Tokyo, Japan) supplemented with 4 ng/mL recombinant basic fibroblast growth factor (WAKO, Osaka, Japan). The mouse C3H10T1/2 cell line that was purchased from RIKEN BioResource Center (Tsukuba, Japan) was cultured in Eagle basal medium (Life Technologies, Carlsbad, CA, USA) containing inactivated 10% FBS and 2 mM L-glutamine (Life Technologies). The human ES cell differentiation medium was Iscove's modified Dulbecco's medium (Sigma-Aldrich) supplemented with an insulin-transferrin-sodium selenite cocktail (Life Technologies), 2 mM L-glutamine, 0.45 mM α-monothioglycerol (Sigma-Aldrich), 50 mg/mL ascorbic acid (Sigma-Aldrich), and 15% FBS.

### MMCT

MMCT was performed as described previously^20^. A9 cells containing hChr.21 were used as donor microcell hybrids. Briefly, WT-ES1 and GATA1s-ES (pZ7) cells were fused with microcells prepared from donor hybrid A9 (21-16) cells and selected with G418 (50 μg/mL). The transferred hChr.21 in each line was characterised by cytogenetic analyses.

### Microarray analyses

Total RNA from WT-ES, Ts21-ES, GATA1s-ES and GATA1s/Ts21-ES cells was prepared using RNeasy (Qiagen, Hilden, Germany) according to the manufacturer instructions. Microarray analyses were performed using a 3D-Gene Human Oligo chip 25k (Toray Industries Inc., Tokyo, Japan). Microarray slides were scanned using a 3D-Gene Scanner (Toray Industries) and processed by 3D-Gene Extraction software (Toray Industries).

### Cytogenetic analyses

Slides of microcell hybrids and ZFN-transfected clones were stained with quinacrine mustard and Hoechst 33258 to enumerate chromosomes. Images were captured using an AxioImagerZ2 fluorescence microscope (Carl Zeiss GmbH, Jena, Germany). FISH analyses were performed using fixed metaphases of microcell hybrids using digoxigenin-labelled (Roche, Basel, Switzerland) human Cot-1 DNA (Life Technologies) and biotin-labelled (Roche) pSTneo plasmid DNA essentially as described previously^20^. Chromosomal DNA was counterstained with DAPI (Sigma-Aldrich). Images were captured using the NIS-Elements system (Nikon, Tokyo, Japan). mFISH analyses were performed in accordance with the manufacturer instructions (MetaSystems, Altlussheim, Germany). Human mFISH probes were purchased from MetaSystems GmbH. Metaphase images were captured digitally with a CoolCubeI CCD camera and the ISIS mFISH software program (MetaSystems).

### Teratoma formation and histology

To produce teratomas, 1 × 10^6^ WT-ES, Ts21-ES, GATA1s-ES, and GATA1s/Ts21-ES cells were subcutaneously injected into testes of SCID mice (Charles River, Yokohama, Japan). After 8 weeks, resected teratomas were fixed in 20% formalin, processed for paraffin sectioning, and then stained with haematoxylin and eosin. All animal experiments were approved by the Institutional Animal Care and Use Committee of Tottori University.

### Transfection

Custom-designed ZFN plasmids and ZFN-encoding mRNA for targeted mutation of the human *GATA1* gene were generously provided by Sigma-Aldrich. The design, cloning, and validation of the ZFNs were performed by Sigma-Aldrich. Human ES cells (2 × 10^6^) were collected in 100 μL Nucleofector solution (Lonza, Tokyo, Japan) with 2.5 μg of each ZFN plasmid and 2.5 μg of pCX-EGFP (gift from Dr. Okabe, Osaka University) or with 2 μg of each ZFN-encoding mRNA and 2.5 μg of pCX-EGFP and were transfected using Nucleofector (Lonza). Twenty-four hours before and after transfection and after sorting, a Rock inhibitor, 10 μM Y-27632, was added to the culture medium. The GFP-positive fraction of the transfected cells was sorted by fluorescence-activated cell sorting 2 days after transfection. Three hundred eighty-four clones using the mRNAs and 96 clones using the plasmids were picked and expanded for further analyses.

### Genomic PCR and mutation analyses

Genomic DNA was extracted from human ES cell lines that were transfected with ZFN plasmids or ZFN-encoding mRNAs using a genomic extraction kit (Gentra System, Minneapolis, MN, USA), and PCR was performed using primers as follows. Primer pairs for the SURVEYOR mutation detection assay (Cel1 assay) and RFLP analyses using BsiHKAI restriction enzyme to detect the mutation in the *GATA1* region were GATA1-F/GATA1-R (347 bp), 5′-TTTCTGTGTCTGAGGACCCC-3′ and 5′-GACCTAGCCAAGGATCTCCA-3′. The Cel1 assay was performed using SURVEYOR Mutation Detection Kits (Transgenomic, Omaha, NE, USA) in accordance with the manufacturer instructions. PCR products were purified by QIAquick PCR Purification Kit (Qiagen), digested with the enzyme, electrophoresed on a 2% agarose gel, and stained with ethidium bromide. Furthermore, the PCR products were subcloned into the pCR4-TOPO vector (Life Technologies), and the vector DNA was sequenced by a 3130xL Genetic Analyzer (Life Technologies) sequencer.

### Haematopoietic differentiation of human ES cells

The differentiation of human ES cells into haematopoietic cells was performed as described previously^30^. In brief, small clumps of human ES cells were transferred onto mitomycin C-treated C3H10T1/2 cells and co-cultured in haematopoietic cell differentiation medium supplemented with 20 ng/mL human vascular endothelial growth factor (R&D Systems, Minneapolis, MN, USA), which was replaced every 3 days. On day 14 of culture, the haematopoietic progenitor cells (HPCs) within the ES-sacs were collected and then transferred onto fresh mitomycin C-treated C3H10T1/2 cells and further cultivated in differentiation medium supplemented with human thrombopoietin (R&D systems) and combinations of other cytokines/mediators (human stem cell factor (R&D systems), heparin sodium (Ajinomoto Pharmaceuticals Co, Tokyo, Japan), and human erythropoietin (Prospec-Tany TechnoGene, East Brunswick, NJ, USA)). On day 17, an equal volume of the medium was added, and cells were further incubated for 3 days.

### Western blot analyses

Protein extracted from the differentiated ES cells was separated by sodium dodecyl sulphate polyacrylamide gel electrophoresis on an 8% polyacrylamide gel and transferred to a polyvinylidene difluoride membrane. The membrane was blocked with 5% dry milk and probed with a mouse monoclonal antibody against BACH1 (F-9 (sc-271211); Santa Cruz Biotechnology, Santa Cruz, CA, USA) or a goat polyclonal antibody against the C-terminus of GATA1 (C20 (sc-1233); Santa Cruz Biotechnology). The membrane was then incubated with a horseradish-peroxidase–conjugated secondary antibody and developed with enhanced chemiluminescence reagents (Pierce Western Blotting Substrate; Thermo, Yokohama, Japan). To confirm that the amount of protein in each lane was comparable, the membrane was stripped and probed with a monoclonal antibody against α-tubulin (DM-1A; ICN Biomedicals, Santa Ana, CA, USA). HEL 92.1.7 whole cell lysate and K562 nuclear extract were used as positive controls (sc-2130 and sc-2277, respectively; Santa Cruz Biotechnology). C3H10T1/2 whole cell lysate was used as a negative control.

### Flow cytometry analyses

The expression of cell surface molecules was analysed by flow cytometry (FACSAria; Becton Dickinson, Franklin Lakes, NJ, USA). On day 14, a fraction of the HPCs within the ES-sacs were stained with CD34 antibody for 30 minutes on ice. Nonadherent cells on day 20 of culture were prepared in PBS containing 3% FBS (staining medium) and stained with combinations of antibodies for 30 minutes on ice. All samples were then washed with staining medium and analysed by flow cytometry. The following antibodies were used: allophycocyanin (APC)-conjugated anti-CD34 (Biolegend, San Diego, CA, USA), APC-conjugated anti-CD41a (integrin αIIb subunit, Biolegend), phycoerythrin (PE)-conjugated anti-CD41a (Biolegend), PE-conjugated anti-CD42b (Glycoprotein Ibα, Biolegend), PE-conjugated anti-CD71 (BD Pharmingen, San Diego, CA, USA), and APC-conjugated anti-CD235 (Glycophorin A, Biolegend).

## Author Contributions

Y.K. participated in all aspects and prepared the manuscript; N.K., Y.Y. and S.Y. performed chromosome transfer and differentiation experiments; K.K. performed cytogenetic analyses; M.Osaki and S.T. performed teratoma formation and histological analyses; H.S. created human ES cell sublines; S.A., K.H. and T.S. performed mutation analyses; and T.T., R.S., H.N., T.Y. and M.Oshimura supervised the study.

## Supplementary Material

Supplementary Information Supplementary information
