# Supplementary material for: Down syndrome-associated haematopoiesis abnormalities created by chromosome transfer and genome editing technologies
Source: Sci Rep. 2014 Aug 27;4:6136. doi: 10.1038/srep06136 (PMC4145315; doi:10.1038/srep06136)
Supplement: Supplementary Information [file srep06136-s1.doc]

**Supplementary** **information**

**Down syndrome-associated haematopoiesis abnormalities created by chromosome transfer and genome editing technologies**

**Yasuhiro Kazuki^1,2*^, Yuwna Yakura^2^, Satoshi Abe^1^, Mitsuhiko Osaki^2,3^, Naoyo Kajitani^2^, Kanako Kazuki^2^, Shoko Takehara^2^, Kazuhisa Honma^1^, Hirofumi Suemori^4^, Satoshi Yamazaki^5^, Tetsushi Sakuma^6^, Tsutomu Toki^7^, Ritsuko Shimizu^8^, Hiromitsu Nakauchi^5^, Takashi Yamamoto^6^ and Mitsuo Oshimura^1,2*^**

^1^Department of Biomedical Science, Institute of Regenerative Medicine and Biofunction, Graduate School of Medical Science, Tottori University, 86 Nishi-cho, Yonago, Tottori 683-8503, Japan; Tel: +81-859-38-6412

^2^Chromosome Engineering Research Center (CERC), Tottori University, 86 Nishi-cho, Yonago, Tottori 683-8503, Japan

^3^Division of Pathological Biochemistry, Department of Biomedical Sciences, Faculty of Medicine, Tottori University, 86 Nishi-cho, Yonago, Tottori 683-8503, Japan

^4^Department of Embryonic Stem Cell Research, Institute for Frontier Medical Sciences, Kyoto University, 53 Kawahara-cho, Shogoin, Sakyo-ku, Kyoto 606-8507, Japan

^5^Division of Stem Cell Therapy, Center for Stem Cell Biology and Medicine, Institute of Medical Science, University of Tokyo, Tokyo 108-8639, Japan

^6^Department of Mathematical and Life Sciences, Graduate School of Science, Hiroshima University, Higashi-Hiroshima 739-8526, Japan

^7^Department of Pediatrics, Hirosaki University Graduate School of Medicine, 5 Zaifu-cho, Hirosaki 036-8562, Japan

^8^Department of Molecular Hematology, Tohoku University Graduate School of Medicine, Sendai 980-8575, Japan

^*^Correspondence should be addressed to M.O. (oshimura@grape.med.tottori-u.ac.jp) and Y.K. (kazuki@med.tottori-u.ac.jp).

Department of Biomedical Science, Institute of Regenerative Medicine and Biofunction, Graduate School of Medical Science, Tottori University, 86 Nishi-cho, Yonago, Tottori 683-8503, Japan. Tel: +81-859-38-6219. Fax: +81-859-38-6210.

**
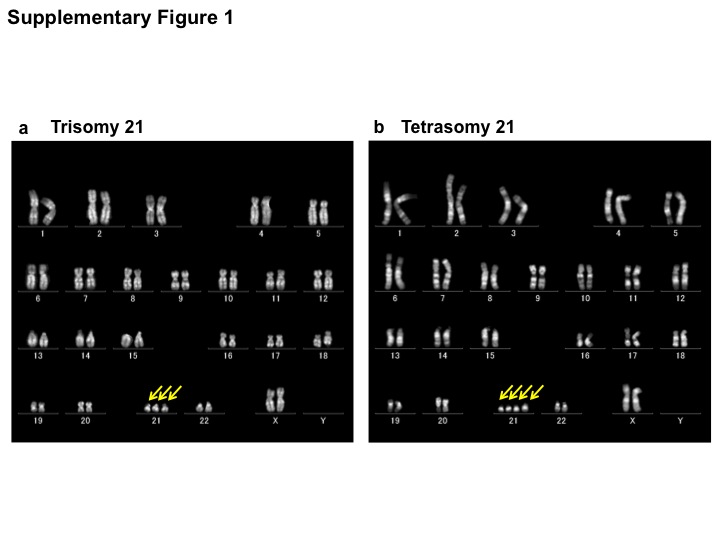
Supplementary Figure 1.** **Cytogenetic analysis of G418-resistant clones after MMCT of hChr.21 to human ES cells.**

Representative karyotypes of trisomy (a) and tetrasomy (b) are shown. Arrows indicate hChr.21.

**
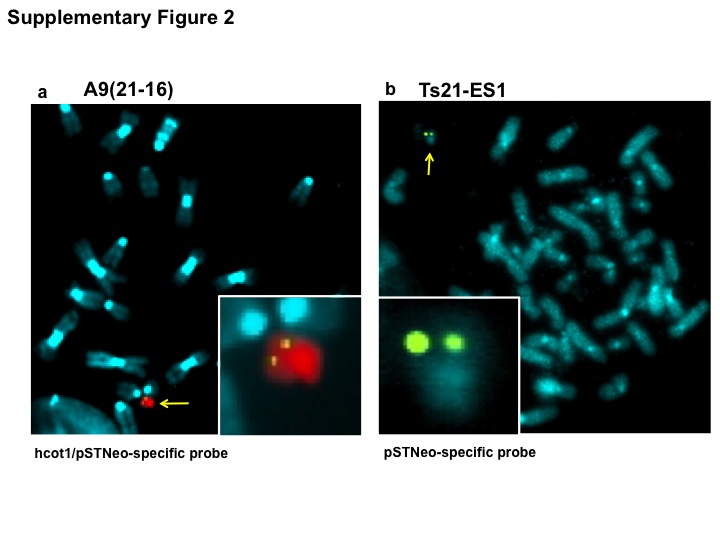
Supplementary Figure 2.** **FISH analyses of A9(21-16) and Ts21-ES cells.**

Metaphase spreads of A9(21-16) (a) and Ts21-ES1 (b) cells by FISH analysis are shown. An arrow indicates hChr.21 tagged with pSTneo, and the inset shows an enlarged image of the hChr.21.

**
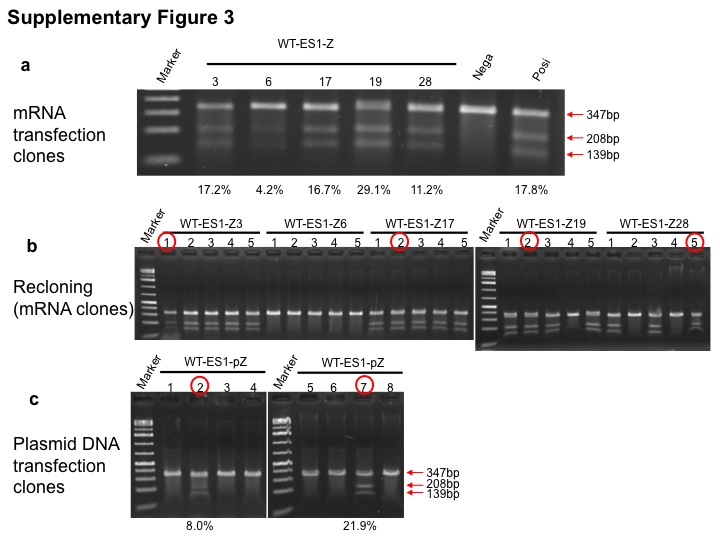
Supplementary Figure 3.** **Cel1 assay of ZFN mRNA- and ZFN plasmid-transfected clones.**

(a) Cel1 assay in ZFN mRNAs-transfected clones. The band intensity was quantified and the ratio between the cleaved and uncleaved bands gives the cleavage rate. The mutagenesis rate induced by the ZFN mRNAs was 4–30%, whereas the negative control (WT-ES cells without the transfection) showed no mutation events, and the positive control (K562 cells transfected with the ZFN mRNAs) showed a 17.8% mutagenesis rate. (b) Cel1 assay of ZFN mRNA-transfected clones after subcloning candidate clones. (c) Cel1 assay of ZFN plasmid-transfected clones. The mutagenesis rate induced by the ZFN plasmids was quantified as described above. Red circles indicate clones used for sequence analyses (b, c).

**
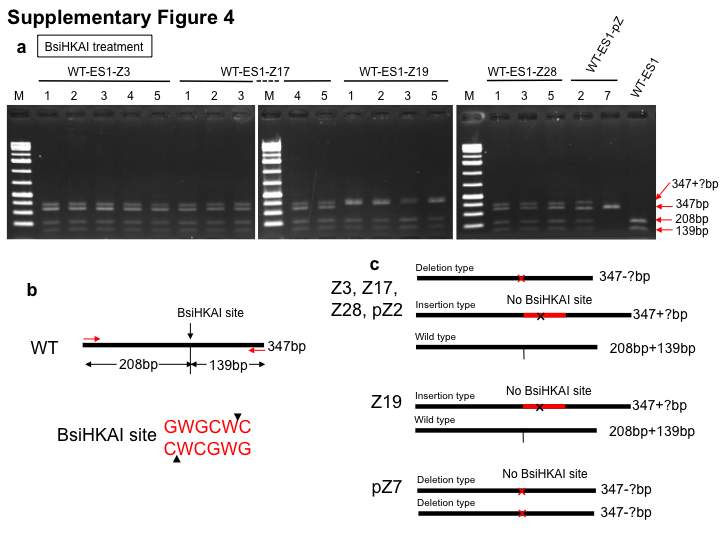
Supplementary Figure 4. RFLP analyses using BsiHKAI.**

(a) Representative data for RFLP analyses using BsiHKAI. (b) Predicted product size with BsiHKAI digestion. (c) Predicted BsiHKAI digestion pattern for GATA1 insertion/deletion mutations in each ZFN-transfected clone.

**
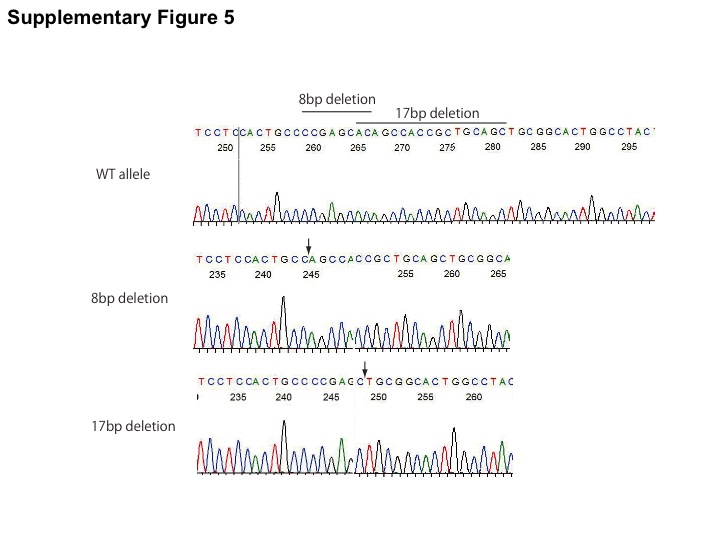
Supplementary Figure 5. Representative sequence data of GATA1s-ES cells.**

The sequence data of representative GATA1s-ES clone (pZ7) is shown. The PCR product of the GATA1 exon 2 region was first cloned into the pCR4-TOPO vector, and 6 independent clones were sequenced. Three clones showed an 8-bp deletion and 3 clones showed a 17-bp deletion. Arrows show break points in each allele.

**
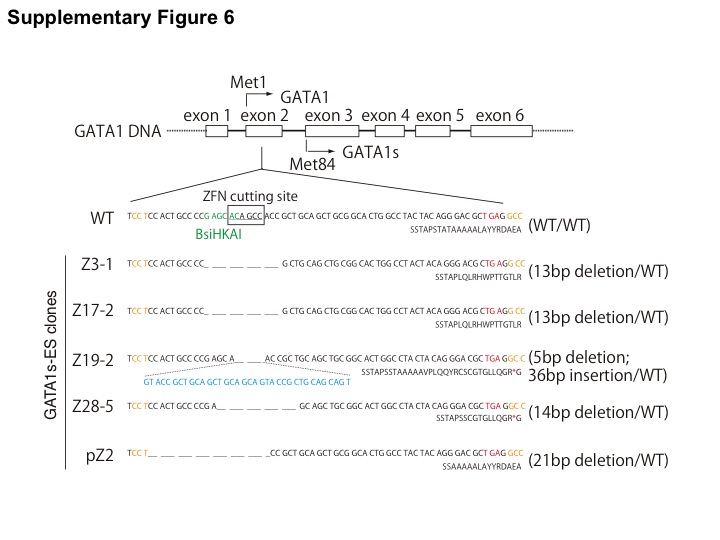
Supplementary Figure 6.** **Sequence analyses of GATA1s-ES cells.**

A deletion and/or insertion within exon 2 of *GATA1* were identified by sequence analyses. Deletion/insertion in Z19-2 and deletion in Z28-5 resulted a TGA stop codon in exon 2 of *GATA1*. An asterisk shows the stop codon. The genomic sequence (upper line) and amino acid sequence (lower line) are shown for each ZFN-transfected cell.

**
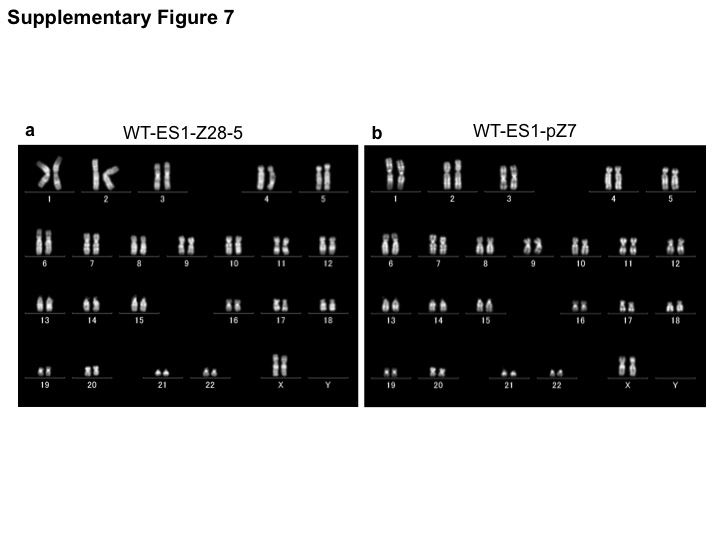
Supplementary Figure 7. Karyotyping of GATA1s-ES cells.**

Representative Q-banding karyotypes of WT-ES1-Z28-5 (a) and WT-ES1-pZ7 (b) cells are shown.

**
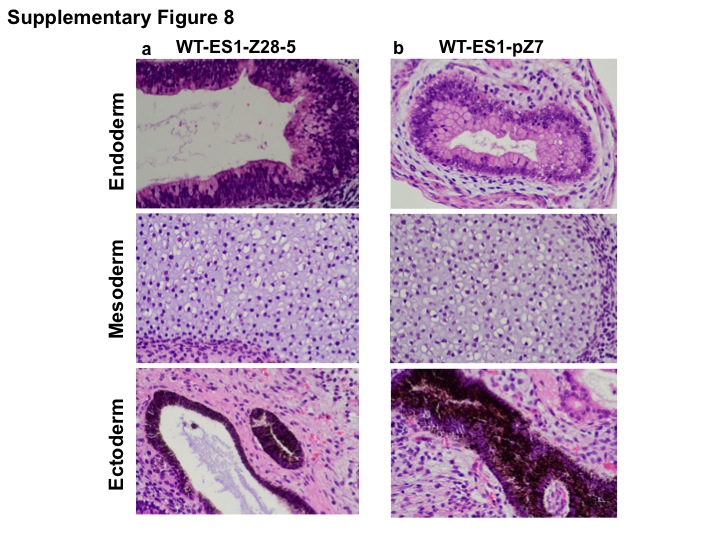
Supplementary Figure 8. Teratoma formation assay using GATA1s-ES cells.**

A teratoma derived from WT-ES1-Z28-5 (a) and WT-ES1-pZ7 (b) cells. Eight weeks after cell transplantation, structures originating from all 3 germ layers were found in the teratoma.

**
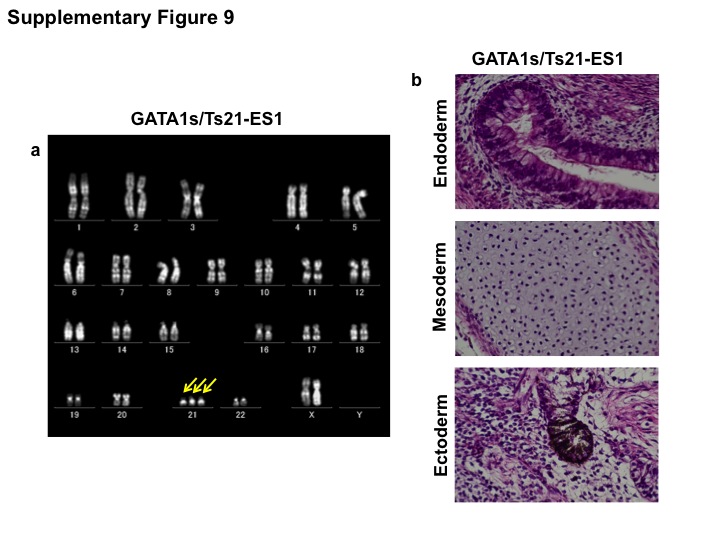
Supplementary Figure 9. Karyotyping and teratoma formation assay using GATA1s/Ts21-ES cells.**

(a) A representative Q-banding karyotype of GATA1s/Ts21-ES cells is shown. Arrows indicate the 3 copies of hChr.21. (b) A teratoma derived from GATA1s/Ts21-ES cells. Eight weeks after cell transplantation, structures originating from all 3 germ layers were found in the teratoma.

**
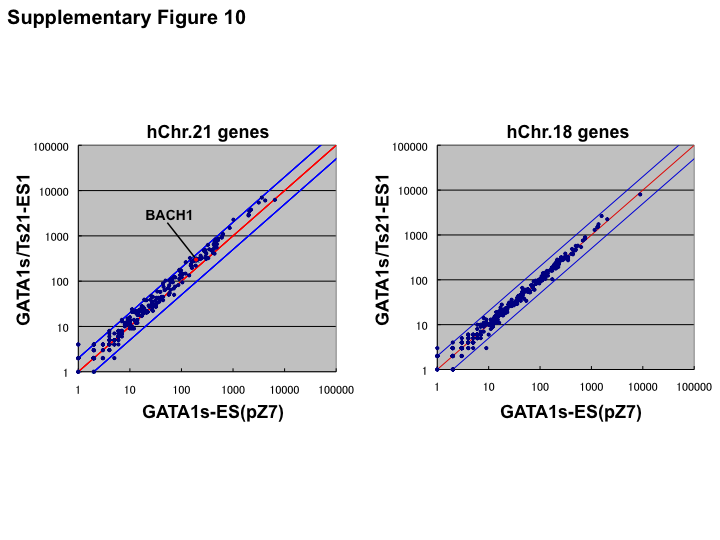
Supplementary Figure 10. Microarray analyses of GATA1s-ES and GATA1s/Ts21-ES cells.**

Representative comparison data of genes on hChr.21 (left panel) and hChr.18 (right panel) between GATA1s-ES and GATA1s/Ts21-ES cells was shown. The dots between blue and red lines and the dots on red line show genes with 2-fold differences in expression and equal expression, respectively. The red dot indicates *BACH1*, a representative gene from hChr.21 (left panel).

**
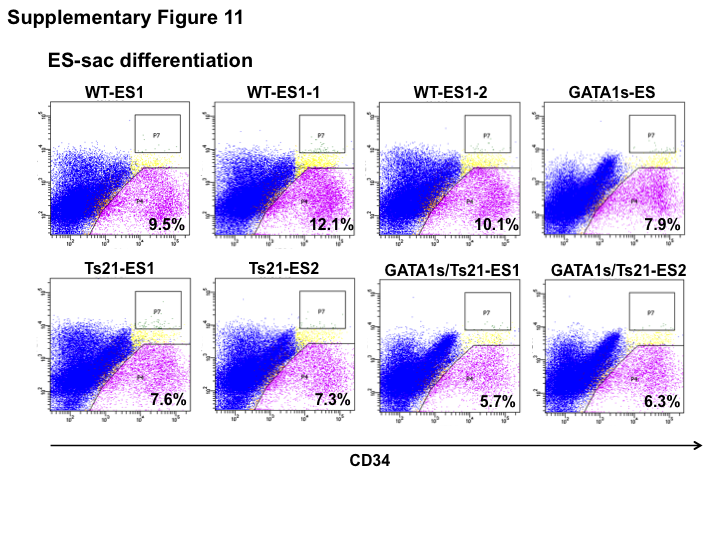
Supplementary Figure 11. Flow cytometry analyses of the ES-sac stage.**

The ratio of CD34-positive cells in the ES-sac stage (day14) is shown. The differentiation ability to hematopoietic stem cells from each ES lines is shown.

**
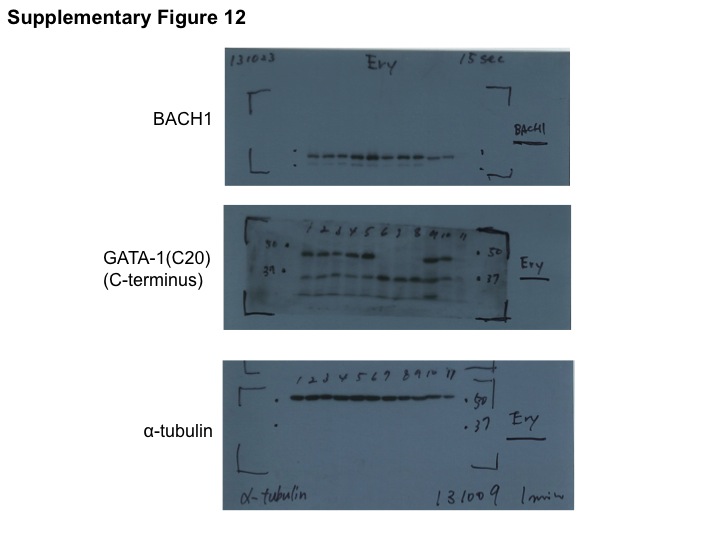
Supplementary Figure 12. Western blot analyses of erythroid lineage-differentiated cells.**

Original full-length blots of erythroid lineage-differentiated cells derived from WT-ES, WT-ES sublines, Ts21-ES lines, GATA1s-ES, and GATA1s/Ts21-ES lines are shown. Lane 1, WT-ES1; lane 2, WT-ES1-1; lane 3, WT-ES1-2; lane 4, Ts21-ES1; lane 5, Ts21-ES2; lane 6, GATA1s-ES (pZ7); lane 7, GATA1s/Ts21-ES1; lane 8, GATA1s/Ts21-ES2; lane 9, HEL 92.1.7; lane 10, K562; lane 11, C3H10T1/2.

**
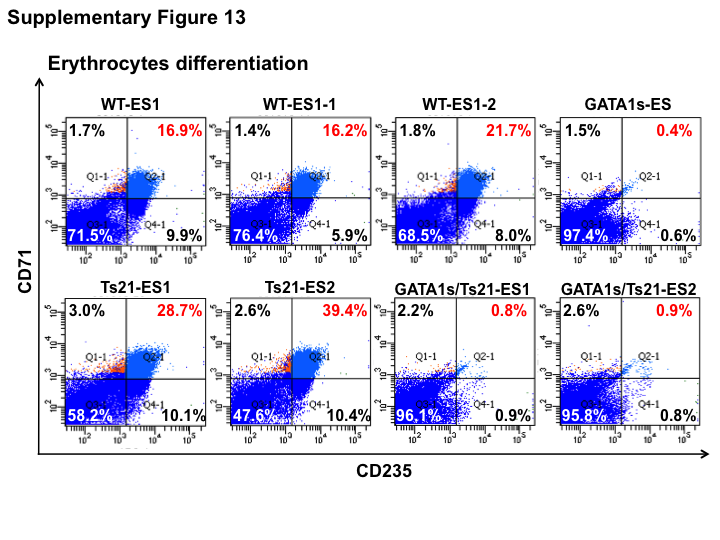
Supplementary Figure 13. Flow cytometry analyses of the ES-derived erythroid lineage.**

The ratio of CD71 and CD235 double-positive cells in the erythroid lineage (day 20) is shown.

**
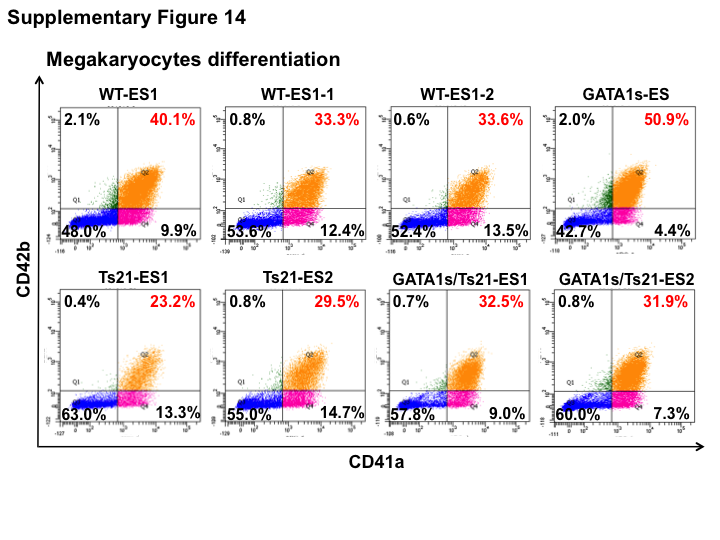
Supplementary Figure 14. Flow cytometry analyses of the ES-derived megakaryocyte lineage.**

The ratio of CD41a and CD42b double-positive cells in the megakaryocyte lineage (day 20) is shown.

**
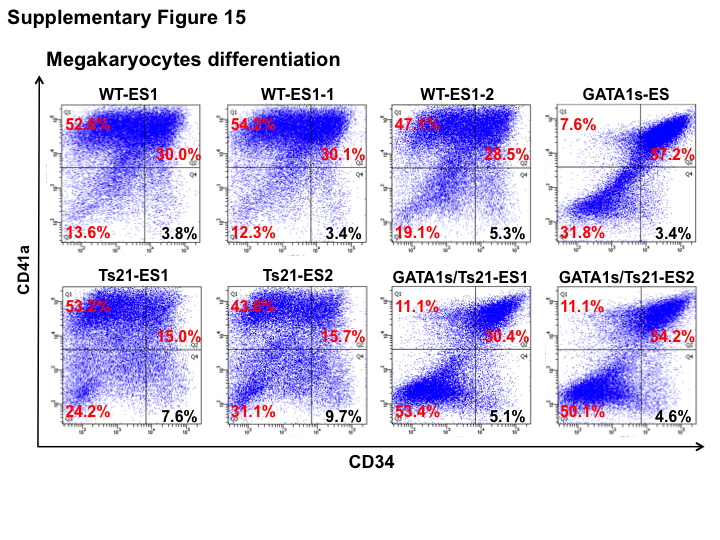
Supplementary Figure 15. Flow cytometry analyses of the ES-derived megakaryocyte lineage using anti-CD34 and anti-CD41a antibody.**

The ratio of CD34-/CD41a-, CD34+/CD41a+ and CD34-/CD41a+ cells in the megakaryocyte lineage (day 20) is shown.
